# BLAST-based structural annotation of protein residues using Protein Data Bank

**DOI:** 10.1186/s13062-016-0106-9

**Published:** 2016-01-25

**Authors:** Harinder Singh, Gajendra P. S. Raghava

**Affiliations:** Bioinformatics Centre, Institute of Microbial Technology, Sector 39-A, Chandigarh, India

## Abstract

**Background:**

In the era of next-generation sequencing where thousands of genomes have been already sequenced; size of protein databases is growing with exponential rate. Structural annotation of these proteins is one of the biggest challenges for the computational biologist. Although, it is easy to perform BLAST search against Protein Data Bank (PDB) but it is difficult for a biologist to annotate protein residues from BLAST search.

**Results:**

A web-server StarPDB has been developed for structural annotation of a protein based on its similarity with known protein structures. It uses standard BLAST software for performing similarity search of a query protein against protein structures in PDB. This server integrates wide range modules for assigning different types of annotation that includes, Secondary-structure, Accessible surface area, Tight-turns, DNA-RNA and Ligand modules. Secondary structure module allows users to predict regular secondary structure states to each residue in a protein. Accessible surface area predict the exposed or buried residues in a protein. Tight-turns module is designed to predict tight turns like beta-turns in a protein. DNA-RNA module developed for predicting DNA and RNA interacting residues in a protein. Similarly, Ligand module of server allows one to predicted ligands, metal and nucleotides ligand interacting residues in a protein.

**Conclusions:**

In summary, this manuscript presents a web server for comprehensive annotation of a protein based on similarity search. It integrates number of visualization tools that facilitate users to understand structure and function of protein residues. This web server is available freely for scientific community from URL http://crdd.osdd.net/raghava/starpdb.

**Reviewers:**

This article was reviewed by Prof Michael Gromiha, Prof. Thomas Dandekar and Dr. I. King Jordan.

**Electronic supplementary material:**

The online version of this article (doi:10.1186/s13062-016-0106-9) contains supplementary material, which is available to authorized users.

## Background

The function and structure of a protein are primarily determined by the amino acid sequence in a given environment. Thus, understanding of sequence function and sequence-structure relationship is necessary to predict structure and function of a protein. Due to the advent of next-generation sequencing, thousands of genomes have been sequenced, and many more are being sequenced. It leads to exponential growth in the size of protein databases. It is nearly impossible to perform annotation or characterization of newly sequenced proteins using experimental techniques. In order to assist the scientific community, numerous computational tools have been developed for annotating proteins from their amino acid sequence. The BioSapiens Network deploys several new methods using the Distributed Annotation System (DAS) for comprehensive prediction of genome and proteome annotation [1–3].

Homology or Similarity-based techniques are routinely used for annotating a protein. In similarity-based approach, we predict structure of a query protein based on its similarity with known proteins whose structure is already known using experimental techniques. There are number of software packages commonly used for similarity search that includes BLAST [4] and HMM [5]. It is well-known fact that similarity-based methods are most successful in case there is a similarity between the query and experimentally annotated proteins [6, 7]. Following are few examples of similarity-based methods and databases; ConFunc, PFP, STRAP, PRSF, BLAST2GO, CDD, PROSITE, Pfam, SMART, ProDom [8–17]. In summary, homology-based methods are imperative in the present era as known proteins (experimentally characterize data) are growing at an exponential rate, the thus probability of getting similar characterize protein is also increasing. One of the major limitations of BLAST is an interpretation of its search result. It easy to perform BLAST against protein databanks (PDB), but it is difficult to perform structural annotation of protein at residue level from the search result. Though number of blast parsers have been developed in past, still residue level annotation of protein is a cumbersome task for a biologist.

In this study, we have developed a similarity-based application StarPDB (Structural Annotation of Residues using PDB) for structural annotation of protein at the residue level. The StarPDB performs BLAST search against customized set of PDB chains, and similar aligned PDB chain regions were used to infer the annotation of query sequence [4, 18]. The structure and function information of the PDB chains were obtained from the ccPDB database [19].

## Methods

The StarPDB web-server has been developed using HTML, JavaScript and PHP 5.2.9 as the front end and installed on a Red Hat Enterprise Linux 6 server environment. The StarPDB web-server consists of three steps: 1) creation of customized databases, 2) annotation using BLAST and 3) visualization of the results.

### Databases of PDB chains for BLAST search

In this study, we derived number of structure or function specific databases of PDB chains from the ccPDB database [19]. In this study secondary structure and accessible surface area information is assigned using DSSP [20]. Every residue assigned surface area is compared with the residue in Gly-X-Gly position [21]. Residues having more than 20 % and less than 20 % accessible surface area are defined as exposed and buried respectively [22, 23]. The β-turn, γ-turn, beta bulge, beta-hairpin and psiloop information is assigned using promotif package [24]. Residues in PDB chains having interaction distance less than or equal to 4.0 Å are consider for annotation. The ligand-protein interaction data is assigned using the Ligand Protein Contact (LPC) package [25]. Interacting residues in PDB chain having distance less than or equal to 4.0 Å are consider for ligand annotation. For each category of the dataset, we created 40, 70 and 100 % non-redundant datasets using the formatdb program in NCBI toolkit. Brief description of various PDB chain databases is given in Table 1. Since, every protein sequence has secondary structure and accessible surface area values for every residue, these two modules share a same dataset. The current release of PDB (July 2015) is used to generate various databases/datasets.

**Table 1:** Brief description of various PDB chains databases and number of protein chains present in respective database

Sub-Database	Software used for assignment	Types of information in database	Number of protein chains		
			Unique	70 %	40 %
SecStructure	DSSP	Regular secondary structure	97216	30642	24211
		Accessible surface area			
BetaTurn	Promotif	Beta Turns and types	90331	27763	21075
GammaTurn		Gamma turns	72124	22971	16584
BetaBulge		Beta bulges in proteins	56186	17753	12944
BetaHairpin		Beta hairpins	72420	21623	15477
PsiLoop		Psi loops	13351	4382	3151
DNAinteract		DNA binding sites	2123	849	757
RNAinteract		RNA binding sites	1437	885	650
LigandInt	LPC	Ligand interacting residues	51615	17952	13525
MetalInt		Metal binding residues	22629	9160	7286

### Sub-databases

As described above server utilizes ten types of sub-databases each containing unique protein chains having specific structure/function. Though these databases are non-redundant but the level of redundancy cut-off is 100 %, it means they may have highly similar sequences, except identical sequences. In a BLAST search, it is possible that the top 10 hits are highly similar and may align against the specific region of the query sequence. In order to overcome this limitation or increasing coverage, we created non-redundant database correspond to our sub-databases based on level of redundancy. In this study, we created two types of non-redundant databases for each sub-database with redundancy level 40 and 70 %.

### Validation dataset

In order to validate the quality of annotation, we created a validation dataset. Our web server StarPDB uses Blast databases generated from PDB of release July PDB 2015. Thus we randomly select around 100 PDB chains from PDB release between August to September 2015. It means PDB chains in validation dataset (used for validation) are different than PDB chains used for prediction in StarPDB are different. In order to evaluate performance of a module we used 10 PDB chains from validation dataset.

### BLAST based annotation

**Fig. 1:**
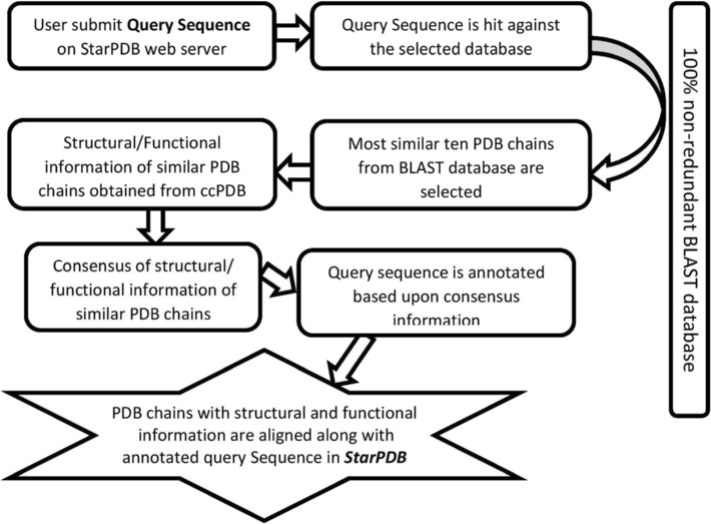
Flow chart shows steps involved in annotation of a query protein at StarPDB server

Several annotation modules are implanted in the StarPDB web server. In all of these modules, we used a similar procedure for annotation; BLAST search against a specific database and parsing of BLAST output. Following is a brief description of steps involved in the annotation (Fig. 1). First, StarPDB performs BLAST search on a query sequence against a user-selected database of protein chains. Second, BLAST output was processed, and ten most similar segments of PDB chains (E-value less than 0.1) were selected for further processing. Third, selected top 10 hits were aligned with query sequence, merged for overlapping alignment to obtain consensus. Finally, query protein was annotated based on alignment and structure/function information derived from ccPDB database. Brief description of annotation procedure used on our server is given in Fig. 1.

### Visualization of annotation

This server displays the annotation of the query sequence and structural/functional information of PDB chains along with matching region information in a grid format. The grid is implemented using the combination of jqxGrid, jqxListBox from jqWidgets package (http://www.jqwidgets.com). The jqxGrid is an advanced data grid for rich visualization and support for client-side editing. It also allows users to change themes for better visualization and export of results in Microsoft Excel format, XML, CSV (comma-separated values), TSV (Tab-separated values) and HTML.

## Results and applications

StarPDB consists of several modules for annotation of the regular secondary structure and irregular secondary structure elements. We have also integrated module for annotation of nucleic acid (DNA/RNA), ligand/metal interaction region/residues. The PDB chain module provides the full annotation of the query sequence against the most similar PDB chain. By default, StarPDB provides annotation information based upon ten most similar PDB chains with E-value less than 0.1. The user can change the number of PDB chains considers for annotation and E-value to increase or decrease the PDB search space. The default matrix used for searching PDB chains is BLOSUM62; the user can select another matrix accordingly. Every annotation module performs the BLAST hit of the query sequence against default 100 % non-redundant (or unique chains) database. Users can also select non-redundant databases at 40 and 70 % sequence similarity level for comprehensive information.

**Fig. 2:**
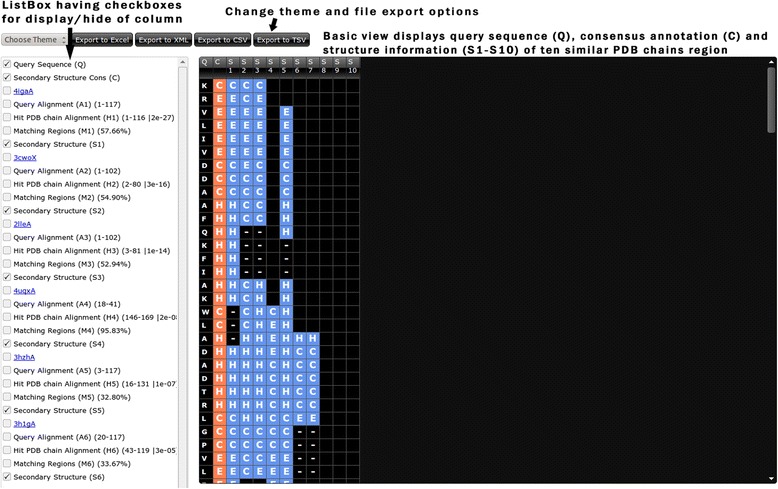
Screen shot of basic view of secondary structure module of StarPDB, checkboxes with options are shown in left column. Query sequence, consensus secondary structure and secondary structure of similar protein chains are shown in right block

**Fig. 3:**
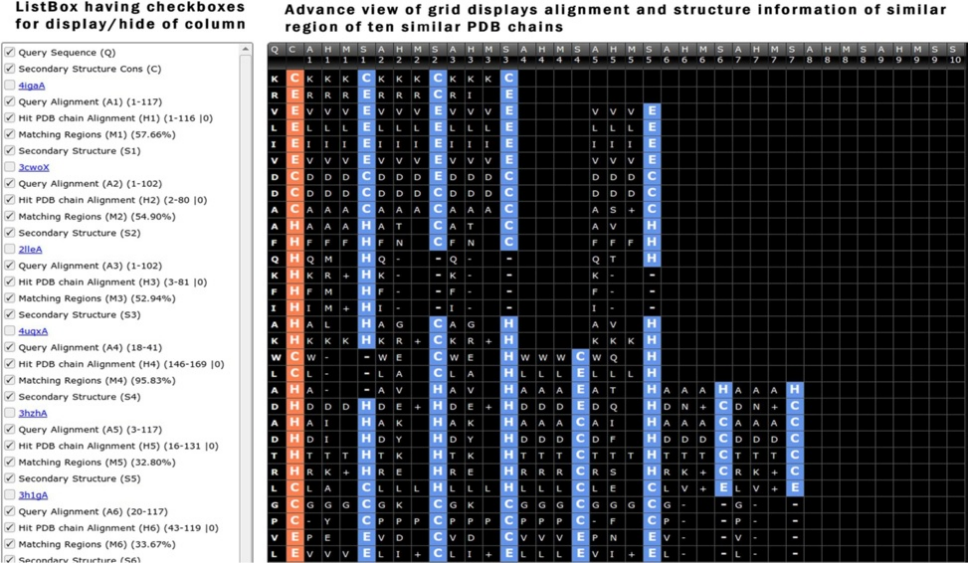
Screen shot of advance view of secondary structure module of StarPDB, checkboxes with options are shown in left column. Query sequence, consensus secondary structure and secondary structure of similar protein chains are shown in right block

The output page of StarPDB web server consists of export buttons, listbox (left section) and results in a grid format (right part). The listbox display the checkboxes for displaying/hiding the various information columns in the grid of similar ten PDB chains in serial order (1–10) based on BLAST E-value. The default display is the basic view of grid, which can be changed into advance view using the checkboxes (Figs. 2 and 3). The first two checkboxes are of the query sequence (Q) and consensus annotation (C) (Fig. 3). Next, five checkboxes consist of most similar PDB chain ID, region of query sequence similar to PDB chain (A1), region of PDB chain similar to query sequence (H1), matching region with identity score in brackets (M1) and structure or function information of the most similar PDB chain (Fig. 3). The next five checkboxes are of second most similar PDB chain and subsequently checkboxes are for remaining similar PDB chains. The advance view of the grid displays the results in columns, the first column is a query sequence with annotation in the second column, and annotated residues are shown in orange color. Next four columns consist of A1, H1 and M1 regions with last column display the assigned structure/function of H1 region of PDB chains in blue color (Fig. 3). The basic view of the grid display only the structure and functional information of PDB chains and excludes the A1, H1, and M1 columns (Fig. 2). The grid allows live editing of all columns including consensus annotation of the query sequence. For offline editing, the user can export the grid in Excel format, CSV, TSV or XML format. We have implemented eight different themes for enhanced visualization of the grid with black as the default theme. Following is a brief description of various structural and functional annotation modules incorporated in StarPDB.

### Regular secondary structure

This module provides the secondary structure annotation of a query sequence based on its similarity with protein sequences in SecStructure database. The annotated secondary structure of protein can be obtained in form of eight states originally obtained from DSSP. These eight states includes three types of helix (G ➜ 3_10_ , H ➜ **α**, I ➜ π ), two types of strands (E ➜ extended strand, B ➜ isolated β-bridge) and coil (T ➜ tight turns, S ➜ Bend, C ➜ Coil) [20]. The default output consists of query sequence (Q), annotation based on consensus of similar PDB chains and secondary structure of similar region of PDB chains (S1–S10) (Fig. 2). The secondary structure of query protein is predicted based on top BLAST hit, remaining residues are assigned based on 2^nd^ BLAST hit and so on (Fig. 3). Using the checkboxes user can display and inspect the matching region and modify the consensus secondary structure accordingly.

### Accessible surface area (ASA)

The ASA module provides the accessible surface area of residues in terms of exposed or buried by performing similarity with protein sequences in SecStructure database. The accessible surface area of protein are obtained using the DSSP and a cut-off of 20 % is used to define exposed or buried residue as stated in method section. The module is similar to regular secondary structure module, except it annotate exposed or buried residues.

### Irregular secondary structure

#### β-turn annotation

**Fig. 4:**
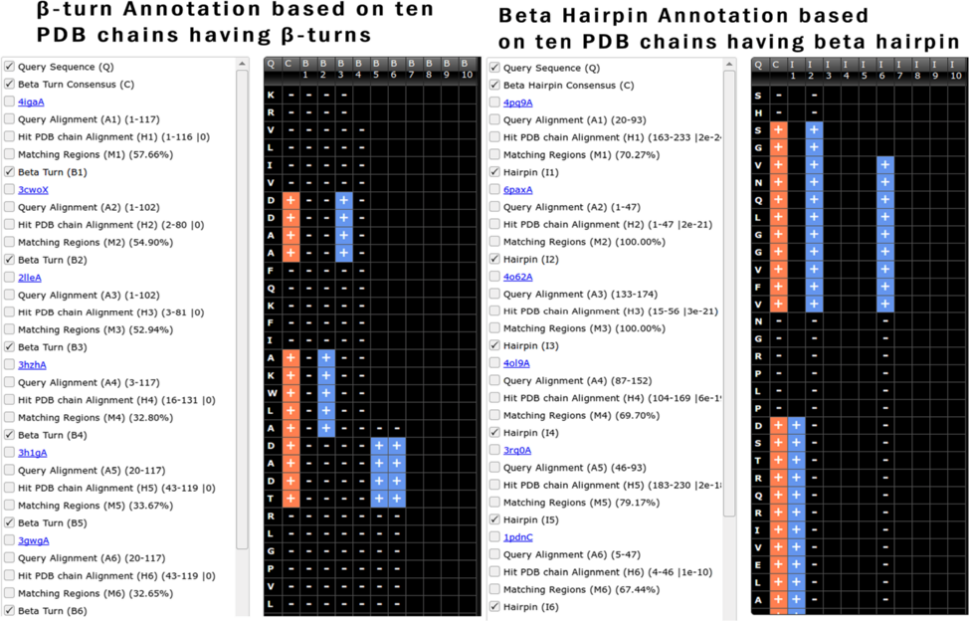
Screen shot of beta turn and beta hairpin annotation module of StarPDB, checkboxes with options are shown in left column. Query sequence, consensus beta turn/beta hairpin and beta turn/beta hairpin of similar protein chains are shown in right block

This module allows users to predict/annotate β-turn and nine types β-turn in a query protein sequence. It is based on similarity of query protein with protein chains in BetaTurn sub-database. Regions of the query sequence annotated by multiple PDB chains have a higher probability of β-turn formation (Fig. 4). Thus users are advised to increase the number of similar PDB chains in the input form. The β-turn types option provides in-depth annotation based upon nine types of β-turn. A careful examination of results must be performed for better understanding of β-turn and types annotation.

#### γ-turn

The γ-turn module helps in the annotation of the potential γ-turns region and their types in the query sequence. This module is similar to β-turn annotation module, except the underlying database is GammaTurn. As compared to β-turn; γ-turn are less abundant in proteins, and the annotation is based upon fewer PDB chains.

#### Beta bulge

This module annotates the potential beta bulge occurrence in a query sequence based upon similarity with proteins in subdatabase BetaBulge. To the best of author’s knowledge, no method is available for prediction of the beta bulge in a protein sequence. The module allows users to either annotate beta bulge or beta bulge types in proteins.

#### Beta hairpin

Annotation of the beta-hairpin in a query sequence is performed using the BetaHairpin database. The ccPDB database holds all the variation of beta hairpins, irrespective of the length of the loop region. The module annotates the complete beta-hairpin region including the beta sheet and loop regions (Fig. 4).

#### Psiloop

The psiloop module predicts the potential psiloop region in a query sequence. The psiloop module is identical in functionality to beta-hairpin, except the underlying dataset is exclusively of psiloop containing PDB chains (PsiLoop database).

### DNA/RNA interacting residues

**Fig. 5:**
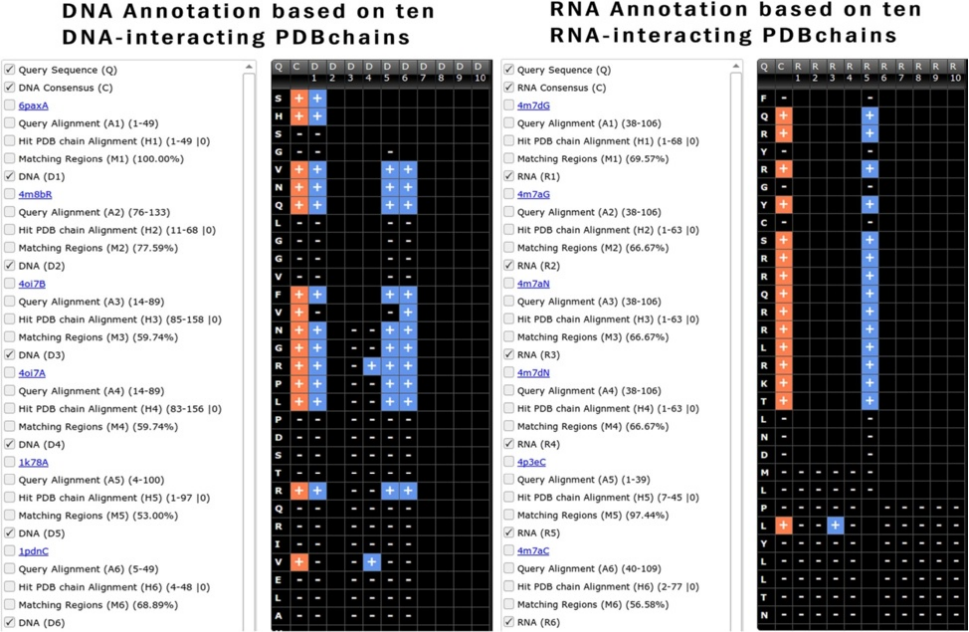
Screen shot of DNA and RNA annotation module of StarPDB, checkboxes with options are shown in left column. Query sequence, consensus DNA/RNA and DNA/RNA interacting residues in similar protein chains are shown in right block

Thousands of proteins interacting with nucleic acid (DNA/RNA) complexes have been deposited in PDB. Thus, similarity search against these complexes helps in better identification of nucleic acid interacting regions or residues in a given proteins. The query sequence is searched against DNAinteract or RNAinteract database for predicting potential interacting regions. The DNA or RNA interacting residues are highlighted with a (+) sign (Fig. 5).

### Ligand/metal and nucleotides interacting amino acid annotation

The term ligand is defined as non-biopolymers that interact with biopolymers (protein and nucleic acids). There are more than 16,000 ligands present in PDB, although many ligands rarely occur in PDB. The ligands module performs the blind prediction of interacting ligands and region in the query sequence. This module annotates query sequence at residue level based on the ligands present in the most similar PDB chains. Thus, users can infer which region of the query sequence has potential to interact with ligands. The user can also select or provide a set of ligands, and the query sequence is annotated based upon the PDB having the selected ligands. For better understanding of the results, a table is displayed in the grid along with the ligand code, its respective name, molecular weight, formula and structure in smiles format (Table 2).

**Table 2:** Brief description of interacting ligands in PDB chain

Ligand code	Ligand formula	Ligand MW	Ligand name	Ligand SMILES
BLA	C33 H34 N4 O6	582.65	Biliverdine IX alpha	Cc1c(c([nH]c1\C=C/2\C(=C(C(=O)N2)C=C)C)\C=C/3\C(=C(C(=N3)\C=C/4\C(=C(C(=O)N4)C)C=C)C)CCC(=O)O)CCC(=O)O
BO3	B H3 O3	61.83	Boric acid	B(O)(O)O
BOG	C14 H28 O6	292.37	B-Octylglucoside	CCCCCCCCO[C@H]1[C@@H]([C@H]([C@@H]([C@H](O1)CO)O)O)O
CYC	C33 H40 N4 O6	588.69	Phycocyanobilin	CC[C@@H]\1[C@H](C(=O)N/C1=C\c2c(c(c([nH]2)\C=C/3\C(=C(C(=N3)\C=C/4\C(=C(C(=O)N4)CC)C)C)CCC(=O)O)CCC(=O)O)C)C
MAL	C12 H22 O11	342.30	Maltose	C([C@@H]1[C@H]([C@@H]([C@H](C@H](O1)O[C@@H]2[C@H](O[C@@H]([C@@H]([C@H]2O)O)O)CO)O)O)O)O
MLR	C18 H32 O16	504.44	Maltotriose	C([C@@H]1[C@H]([C@@H]([C@H]([C@H](O1)O[C@@H]2[C@H](O[C@@H]([C@@H]([C@H]2O)O)O[C@@H]3[C@H](O[C@@H]([C@@H]([C@H]3O)O)O)CO)CO)O)O)O)O

**Fig. 6:**
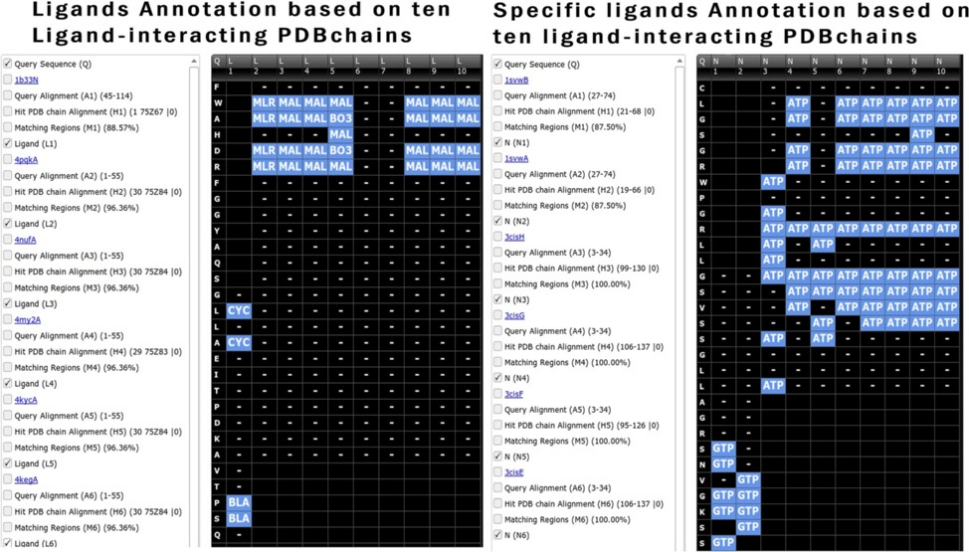
Screen shot of ligand and specific ligand annotation module of StarPDB, checkboxes with options are shown in left column. Query sequence, ligand name and interacting residues in similar protein chains are shown in right block

The ligands module is the only method that can annotate all the interacting ligands in PDB for a given query sequence. An example output is shown in Fig. 6, with a query sequence, alignment region and region of PDB chain annotated with ligand interacting residues. It displays the ligand code at the corresponding interacting residue position. The metals module is identical to ligands module. However, it provides the annotation of potential metals interacting region. A dedicated module for annotation of nucleotide molecules including ATP, CTP, GTP and UTP is also developed (Fig. 6). The ligand and specific ligand interaction annotation module, display the query sequence along with aligned similar PDB chain and corresponding interacting ligand code in blue color.

### Annotation based on single PDB chain

**Fig. 7:**
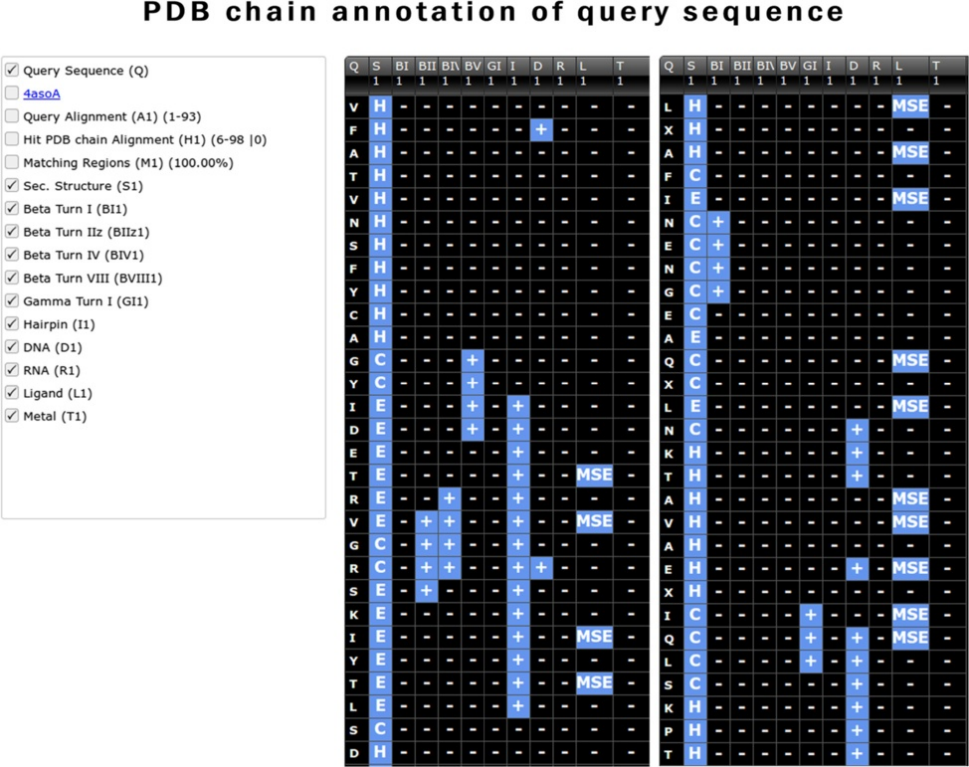
Screen shot of PDB chain annotation module of StarPDB, checkboxes with options are shown in left column. Query sequence and various annotations of PDB chain are shown in right block

Analysing the similar PDB chains using a secondary structure, irregular secondary structure elements, DNA/RNA and ligands/metals interacting modules, the user, can identify the most related PDB chain. The PDB chain module aligns the query sequence against a given PDB chain and displays the alignment with structure information. This module predict complete structural and functional information of each residue in query protein; it includes secondary structure (e.g., Helix, β-turns, γ-turns, beta-hairpin) and functional information (DNA/RNA, ligand/metal interacting residues) based on alignment (Fig. 7).

### Quality of annotation

First, we submit 10 PDB chains to secondary structure and ASA module of StarPDB. In order to evaluate performance we compare predicted and actual secondary structure as well as accessible surface area (Additional file 1: Table S1). As shown in Additional file 1: Table S1, we achieved average secondary structure prediction accuracy 61.07 % with minimum accuracy 43.23 % (PDB chain 4u49A). Similarly we achieved average accuracy 77.79 % in case of accessible surface area with minimum 54.76 % and maximum 96.03 %. We also evaluated turn prediction modules and achieved maximum average accuracy 95.05 % for beta turns and minimum average accuracy 57.22 % for gamma turns (Additional file 1: Table S2). In case of ligand and metal module, we predicted the binding sites of specific ligand and metals and achieved an average accuracy of 41 and 70 % respectively (Additional file 1: Table S3). The details results of the modules along with PDB chain and ligand/metal name are given in Additional file 1: Tables S1, S2 and S3.

## Discussion and conclusion

The annotation of structure and function of the unknown protein is one of the biggest challenges in bioinformatics. In past number of methods have been developed for performing residue level annotation of proteins with high accuracy using knowledge-based and novel techniques. In addition, there is a significant development in similarity search techniques [8, 17, 26]. This raises question why there is need for developing simple BLAST based server for annotation of protein. Bioinformatics scholars are interested in developing advanced techniques for better annotation. Despite BLAST have been developed two decades back and has been citeed by ~54,000 research artilces, it is difficult for a biologist to annotate a query protein at residue level using BLAST based search against PDB. One may argue that it is a trivial job for a bioinformatics scholar to annotate a protein at residue level, but we should understand it is difficult for a biologist; who actually require residue level annotatio. In this study, we make a systematic attempt to facilitate a biologist in assigning structure or function to their protein at the residue level.

Our server has a series of modules for performing comprehensive annotation of protein. The default annotation is based on the consensus of structure/function information of most similar ten PDB chains. The structure/function information of PDB chains is derived from the ccPDB database and non-redundant databases are created using the NCBI toolkit. The number of PDB chains can be increased to boost the annotation confidence score and PDB search space. Since, many PDB chains are similar to each other; the user can select the various non-redundant databases to increase the annotation coverage in PDB. The ligands annotation module is the only method able of annotate all the ligands present in PDB. It also allows users to annotate their query sequence against a specific ligand or a set of ligands. Using the structure and function modules, the user can decide the most related PDB chain and better understand the query sequence structure and interacting region using the PDB chain annotation module. In order to provide a rich visualization environment, we have integrated jqxWidgets.

In this study, we created ten databases for performing BLAST search, one database for each type of structure or function annotation. One may raise the question why we created specific structure/function databases instead of searching against the whole PDB. It is because various structure/function related PDB chains are not equally distributed in PDB, for example, there are limited DNA interacting PDB chains. It is possible that DNA interacting chains or regions are not in top hits if we perform PDB wide BLAST search. In our DNA annotation module, we perform BLAST search against only DNA interacting protein chains. This will allow us to annotate DNA interacting region despite their distribution in PDB is rare. These ten type of databases used in our server allow the user to perform unbiased annotation.

This StarPDB allows the user to perform similarity search against protein chains at different level that includes redundancy level of cut-off 100, 70 and 40 %. This is important to understand why we used three level of redundancy instead of performing BLAST search against 100 % non-redundant database. By default, server performs search against specified non-redundant database at redundancy level 100 % (unique protein). This database of unique protein chains has advantage as it does not contain any identical protein chain, so identical hits will be removed that will improve performance. Though our database of identical protein chain removes all identical chains still, it contains highly similar protein chains. It is possible that top ten similar PDB chains may annotate only a specified region of the query protein and fail to annotate whole query sequence. In order to overcome this limitation, BLAST search against diverse PDB chains will increase the PDB search space and annotation coverage of query sequence. We allow users to perform BLAST against non-redundant datasets at 70 and 40 %, which contains diverse class of PDB chains. We advised users, first they should perform a search against non-redundant at level 100 % if they fail to annotate whole regions than they should try redundancy at 70 or 40 %. StarPDB is a unique resource for the biologist to annotate; edit and analyse structure and functional aspects of their proteins.

## Reviewers’ comments

### Response to Prof Michael Gromiha

Reviewer 1. In this work, the authors developed a tool for structural annotation of a protein based on its similarity with known protein structures using standard BLASTP. It provides information on various factors such as secondary structure and binding sites. It will be useful for scientific community for obtaining structure based features along with alignment.

Comment: 1. For several cases, there are no hits. In such cases, links could be given for predictions (or provide predicted results), for example binding sites.

Response 1: *As suggested by reviewer, we provide a list of non-similarity based prediction methods (See**http://crdd.osdd.net/raghava/starpdb/links.php**); user can use these methods in case of no hits*.

Comment: 2. Secondary structure and ASA can be simultaneously obtained from DSSP. ASA may be included in the list of features.

Response 3: *A new module ASA has been integrated in StarPDB as suggested by reviewer.*

Comment: 3. Table 1 shows the structural classification of proteins from PDB. If feasible, the data may be given for Uniprot sequences, i.e. number of sequences will have beta bulges, DNA binding etc. as per the present similarity search.

Response 3: *It is not possible to provide such analysis on proteins in Uniprot as structures of most of these proteins are not solved.*

### Response to Prof. Thomas Dandekar

Reviewer 2. Dear authors, as already given in the summary, you provide here something potentially quite useful for the community. You now should make sure that the quality of your tool is convincing. There are 2 main concerns: a) maintenance of the server -- who will maintain it, for how long, will for instance updates of PDB be considered? An ad hoc standard is to guarantee this for at least two years (e.g. an NAR policy). Please clearly state here your efforts in this direction. b) Quality of the predictions -- please give some indication how reliable your predictions are, best you compare this to standard techniques for each of the properties and compare the prediction result to specific servers on that property. remark: Actually, for your purpose it is also completely sufficient if you run 100 pdb structures (unbiased selection) through the server and compare the quality of your predictions to the results known from pdbsum regarding these different qualities. It is completely fine, if in some features the quality (just by similarity search) is not particularly high, you are expressively invited to tell that the reader, it does no harm, he only has to know this if he wants to use your nice and simple annotation server.

Similarly, you should make transparent your constructed database for each property and how this affects the quality of the predictions obtained (simple example: if you have low representation of beta-sheet proteins, probably the server will not be strong in recognizing here the right properties and of course you can only deal with structures similar to PDB etc.)

Response: *We agree with reviewer that maintenance this type of servers is costly and complex. Most of groups or institute fail to maintain this type of services, following is point-wise-point response to reviewer’s queries.*

a) Regarding maintenance of the server, our group will maintain this server. Our group developed and maintain more than 100 databases and servers, some of servers are more than 10 years old. This web server is based on ccPDB database, which was developed and published in 2011. We are regularly updating the ccPDB database and is updated with the latest release of PDB (updated up to 30^th^ September). We do maintain all of the servers that we develop in our group.

b) Regarding, quality of the predictions, this server is based on similarity so accuracy will depend on similarity between query and target sequence. As suggested by reviewer we randomly selected around 100 PDB chains (10 chains to evaluate a module) from the PDB release between August and September 2015. None of them included in blast database of StarPDB used for prediction (StarPDB is based July 2015 release of PDB). We submitted 10 PDB chains to different modules of StarPDB in order to predict their secondary structure contents. We compare predicted and actual structure of these PDB chains in order to evaluate the performance of different modules. As shown in Additional file 1: Tables S1, S2 and S3), we achieved average accuracy from 57 to 95 % for modules developed for predicting secondary structures, accessible surface area and backbone structure. Similarly, we achieved average accuracy from 41 to 70 % for modules developed for predicting ligand binding sites e.g., DNA, RNA, metals). As shown in Additional file 1: Tables S1, S2 and S3, there is lot of variation in performance of our modules from 0 to 100 % on different PDB chains depending on level of similarity.

The number of unique PDB chains in sub-databases used by different modules of StarPDB is shown by Table 1. It has been observed that performance depend number of chains used prediction, higher the number of PDB chains in a sub-database higher is the probability of correct prediction. In addition accuracy or correct prediction depend on local similarity or segment similarity between query and target sequence. Thus to facilitate users in understanding segment similarity, we provide similarity details that includes p-value, alignment along with matching regions.

## Second revision comments

Comment: Dear Authors, essentially you gave answers to my reviewer comments. Thank you for revising your paper accordingly. May be you should also stress then where your method excels and where not. Furthermore, do you think 10 pdb structures offers enough for verification? Finally, maintaining several server is some work, but 100 is incredible high, how do you manage?

Response: *Since, StarPDB is a similarity based method, its performance depends on the level of similarity between query and target sequence. Thus our server excels in case query protein have high similarity with proteins whose structure information is available in PDB. Regarding verification based on 10 PDB structure for each module, in this study we are not claiming new method so verification is not required. We are facilitating users in annotating their proteins based on sequence similarity with known structure available in PDB. Validation or verification of similarity based method BLAST is established and beyond of the scope of this study. We demonstrate the performance of our annotation approach using 10 PDB structures for each module. In simple words verification based on 10 PDB structures is not sufficient.*

Thanks for appreciating our effort to maintain more than 100 servers. I am a computer professional responsible to maintain IT department of this institute.

### Response to Dr. I. King Jordan

Reviewer 3. We tested the functionality and utility of the StarPDB webserver using a number of different sequence similarity searches. We found several major issues that mitigate the utility of the tool and should be addressed before the tool is published and widely released. We also describe a number of minor issues that if addressed should enhance the usability of the tool.

Comment 1: Major issues: 1. Since the tool is web-based, it is important for users to have access to a local copy of their results. The tool allows users to export their results in a number of different formats: Excel, XML, CSV and TSV. However, the export tool did not correctly output the results of our searches. For example, irrespective of the output format chosen, blank spaces are filled with “12/31/1969” (Unix time zero minus 1?). In addition, the entire set of results shown in the webserver (i.e. all columns) do not appear in the export files.

Response 1: *As suggested by reviewer, we improved output module of StarPDB. The data is export using the free version of jQWidgets, which allow to export files up to 2 MB size. Thus, any file with size larger than 2 MB failed to export, which happen only with large amount of data while exporting an excel format file.*

Comment 2: The PDB Chain module does not work for all PDB IDs that correspond to sequences with homology to a query sequence found in other modules. For example, when the query sequence RAMP-1 (GI: 6119625) is run through the Tight Turns Beta Turn module, the second best PDB hit is 4rwgA. Visual inspection shows clear similarity between RAMP-1 and 4rwgA. Nevertheless the PDB Chain module did not find any matches. The PDB Chain module does however work for a hit with lower sequence similarity for this same query 2yx8A.

Response 2: *We are thankful to the reviewer for pointing out the problems, we have fixed the problem accordingly. Now the PDB chain module display the result of all PDB chains including the 4rwgA.*

Comment: 3. When clicking on a PDB hit chain, such as 3n7pD, the user is directed to a page that attempts to show the tertiary structure of the selection, but the structure does not display for any PDB hits – the error is always: java.io. FileNotfoundException. This utility apparently uses the IcedTea plugin for Java, and it fails to work on several different browsers (Chrome/Firefox).

Response 3: *This is a standard issue with the java plugins, either they require permission from user or they become obsolete due to java security permission requirement. Hence, we removed the java plugin and redirects the user to the main site PDB for the visualization of protein and other information.*

Comment: 4. In the DNA/RNA module, some resulting hit PDB chains are duplicated: a zinc finger protein query (GI: 212514646) returns 10 hits for both DNA and RNA, all of which were the same PDB chain (2i13A for DNA, 1un6C for RNA). Similarly, a smaller RNA binding protein query (GI: 654101160) returns 4/10 of the resulting RNA hits from the same PDB chain (2errA and 2fy1A).

Response 4: *The StarPDB webserver selects the similar PDB chain region according to BLAST output. Based on the level of similarity different regions of a same PDB chain have different alignment in BLAST output. This usually happens when there is a large gap between two regions having similarity to user given sequence.*

Comment: Minor issues: 1. The menu on the homepage differs compared to the menu when in one of the other pages. On the homepage, one of the options is “Complete” and while on any other page, it is “PDB Chain.”

Response: *We are thankful to the reviewer for the suggestion and we have incorporate them in the StarPDB webserver. We have uniform the menu options across all pages of the StarPDB webserver.*

2. The homepage screenshot example has an “Export to HTML” option, but only the PDB chain module has this option. On this module, the “Export to HTML” option does not match the rest of the export option’s button style, and it does not function.

Response: *The “Export to HTML” option is non-functional due to size limit of exported HTML files. Hence, we have removed this option across StarPDB webserver.*

3. If the PDB chain hits are longer than the query, the resulting table will contain results that extend beyond the query sequence, instead of producing a table that terminates at the end of the query.

Response: *We are thankful to the reviewer for the suggestion, now the table terminates at the end of the query.*

4. On the highest settings (E-value 2000 and 40 blast hits) some of the results do not contain a region that aligns with the query but is still incorporated into the resulting output table.

Response: *We have incorporated a filter in StarPDB that filter out the PDB region having no information for annotation.*

5. When displaying the PDB chain hit alignments, it is not immediately clear which residues align to the query. This requires the user to go through each residue and check to see if it aligned to the hit.

Response: *For viewing the BLAST alignment, user has to check the required checkboxes in the left panel. We have added a statement in the text above result section, that the left panel checkboxes display the BLAST alignment.*

6. PDB chain module only allows for comparison against one PDB chain hit. A nice addition would be a feature in the other modules that allows you to take the resulting hits and run them through a multiple PDB chain version of this module.

Response: *The reviewer has suggested a nice feature and will be useful for annotation through an iterative process. We will incorporate this feature in a future release of StarPDB webserver.*

In order to facilitate the authors’ trouble shooting and improvement of the tool so that the issues enumerated above can be more readily addressed, we provide here a narrative description of the tests that we ran on the different modules of the tool along with the specific issues that were encountered at each step. The specific major and minor issues are cited throughout the narrative as Ma_* and Mi_* respectively. We are including this in the Minor issues part of the web form so that it can be removed from the final review. Description of the StarPDB webserver testing The homepage consists of a menu at the top that allows the user to navigate to each module.Mi_1 This is followed by a short summary of the web-server, and an example screen shot of the expected output.Mi_2 For each module, the query form consists of a submission form to enter a protein sequence in FASTA format, as well as several options to adjust the default settings or use advanced options. For each of these modules, my query was the RAMP-1 Mus musculus protein sequence (GI: 6119625), with the exception of the DNA/RNA module, in which I used krueppel c2h2-type zinc finger protein (GI:212514646) and a RNA binding protein (GI: 654101160) from Tetraselmis. Secondary-Structure Starting with the Secondary-Structure Annotation module with default settings, the results consisted of a table portraying residues corresponding to coil and helix locations. The hit PDB chains were longer than my query sequence, so the resulting table contained information that went beyond my query sequence. Mi_3 Upon attempting to export the results in any format, the resulting file contained dates in several blank spaces, potentially a parsing/Unicode error, and did not contain the same results as the module on the web-server. This export issue was common throughout all export options (excel, xml, csv, tsv) and appeared to be the case for every module.Ma_1 In order to test the limitations of the module, I repeated this search using the highest settings. Hit chains 4 and 9 did not contain any region that aligned with my query, but were still presented as results in the output.Mi_4 Tight Turns The Tight Turns module consists of more irregular secondary structures. There is a submenu allowing the user to select which major secondary structure they want to predict for. It appears that the only way to check for all of these irregular secondary structures is to either run your query through each subcategory, or to check your query against a PDB hit chain in the PDB Chain module. I attempted this using my query and the PDB chain “4rwgA,” which was the second hit (with 65 % matching region) from the Beta Turns module, but did not return any results.Ma_2 DNA/RNA I used a different query protein than RAMP-1 as I was initially not getting any hits. With the zinc finger protein, I got 10 hits for both the DNA/RNA binding prediction, but all 10 hits were for the same PDB chain (2i13A for DNA, 1un6C for RNA). To repeat this, I used a smaller RNA binding protein (GI: 654101160) and 4/10 of the resulting RNA hits were the same PDB chain (2errA and 2fy1A). Ma_4 Ligand/Metals: Using the RAMP-1 Mus musculus protein, I used the ligand interaction search to see that the closest hits bind with the ligand Selenomethionine (MSE). In an attempt to see where the PDB chain hits aligned with my query, I selected each hit to display on the table. It was not easy to determine which residues aligned with my query, and required me to look at each residue of each hit and compare it to the query. When there are many hits on the table, this can become quite cumbersome. Mi_5 PDB Chain: I used the same RAMP-1 query with the PDB chain hit 2yx8A. My only suggestion for this module would be the possibility of adding more PDB chains against which to align the query; perhaps a feature in the other modules that allows you to take the resulting hits and run them through a multiple PDB chain version of this module. Mi_6.

Response: *We are grateful to reviewer for performing in depth analysis on our web server and sharing procedure and results of analysis. This depth analysis help us to fix bugs in StarPDB server.*

## Additional file

Additional file 1: Table S1. STARPDB webserver annotation accuracy of secondary structure, accessible surface area, DNA and RNA modules. **Table S2.** STARPDB webserver annotation accuracy of tight turns modules. **Table S3.** STARPDB webserver annotation accuracy of ligand and metal modules. (DOCX 21 kb)

## Abbreviations

ATP: adenosine triphosphate
BLAST: basic local alignment search tool
CTP: cytidine triphosphate
DNAinteract: DNA interacting PDB chains database
GTP: guanosine triphosphate
LigandInt: ligands interacting PDB chains database
LPC: ligand Protein Contact
MetalInt: metals interacting PDB chains database
PDB: protein data bank
RNAinteract: RNA interacting PDB chains database
SecStructure: secondary structure database
StarPDB: structural annotation of residues using PDB
UTP: uridine triphosphate
β-turn: beta turn
γ-turn: gamma turn
