# Supplementary material for: BLAST-based structural annotation of protein residues using Protein Data Bank
Source: Biol Direct. 2016 Jan 25;11:4. doi: 10.1186/s13062-016-0106-9 (PMC4727276; doi:10.1186/s13062-016-0106-9)
Supplement: Additional file 1: Table S1. — STARPDB webserver annotation accuracy of secondary structure, accessible surface area, DNA and RNA modules. Table S2. STARPDB webserver annotation accuracy of tight turns modules. Table S3. STARPDB webserver annotation accuracy of ligand and metal modules. (DOCX 21 kb) [file 13062_2016_106_MOESM1_ESM.docx]

# **Supplementary Data**

# **BLAST-based structural annotation of protein residues using Protein Data Bank**

Harinder Singh and Gajendra P. S. Raghava*

Bioinformatics Centre, Institute of Microbial Technology, Sector 39-A, Chandigarh, India

**Table S1:** STARPDB webserver annotation accuracy of secondary structure, accessible surface area, DNA and RNA modules.

| **Secondary Structure** | | **Accessible Surface Area** | **DNA** | | **RNA** | |
| --- | --- | --- | --- | --- | --- | --- |
| **PDBID** | **Accuracy** | **Accuracy** | **PDBID** | **Accuracy** | **PDBID** | **Accuracy** |
| 4uf2A | 62.22 | 59.26 | 3x1sC | 100 | 4ue5E | 0 |
| 4xlnE | 61.96 | 86.96 | 5ddgA | 9.09 | 5detA | 63.64 |
| 4z2tA | 51.43 | 66.67 | 3x1vH | 90.91 | 4ue5C | 28.57 |
| 4wu8C | 77.14 | 98.1 | 4xr2A | 100 | 5bymA | 80 |
| 5d1iA | 56.9 | 66.09 | 3x1vB | 100 | 4ue5D | 100 |
| 5d12B | 43.25 | 54.76 | 4zpkA | 100 | 4ue5F | 100 |
| 4u49A | 43.23 | 59.67 | 5d3gD | 0 | 5a0tB | 20 |
| 5a7xM | 76.06 | 94.74 | 4xegA | 100 | 4ue4C | 100 |
| 5ac92 | 60.68 | 95.63 | 4zkgA | 100 | 5detB | 69.23 |
| 5dhfB | 77.78 | 96.03 | 4rsbC | 0 | 4ue5D | 100 |
| **Average** | **61.07** | **77.79** |  | **70.0** |  | **66.14** |

**Table S2:** STARPDB webserver annotation accuracy of tight turns modules.

| **Beta Turn** | | **Gamma Turn** | | **Beta Bulge** | | **Beta Hairpin** | | **Psiloop** | |
| --- | --- | --- | --- | --- | --- | --- | --- | --- | --- |
| **PDBID** | **Accuracy** | **PDBID** | **Accuracy** | **PDBID** | **Accuracy** | **PDBID** | **Accuracy** | **PDBID** | **Accuracy** |
| 5a9eA | 75.86 | 2n7hA | 22.22 | 5bv0A | 11.11 | 5cvjA | 100 | 4wglA | 100 |
| 5c47A | 95.29 | 5bxbA | 50 | 4zinA | 100 | 4z2tA | 97.14 | 5cm7B | 50 |
| 4zinA | 100 | 4whfA | 100 | 4xcaD | 16.67 | 4s3jC | 86.49 | 5brtA | 95.65 |
| 4ztuB | 92.05 | 3jafA | 100 | 4z70A | 88.89 | 4zpiA | 9.18 | 5c4fA | 100 |
| 4z70A | 100 | 5bs2A | 0 | 4reeB | 100 | 4ymbB | 100 | 4z70A | 100 |
| 4qh6A | 100 | 4y9bB | 100 | 4qh6A | 100 | 4wnjB | 100 | 4zbaA | 100 |
| 3jafA | 95.4 | 4qhuC | 100 | 3x42A | 100 | 5bvoA | 98.25 | 4xepA | 100 |
| 3x3xA | 97.81 | 4uw8A | 0 | 4yw6A | 100 | 4uimB | 100 | 4z6lB | 100 |
| 4wepB | 94.12 | 5d16A | 0 | 4zphD | 88.89 | 5bouD | 100 | 5dulD | 100 |
| 4yw6A | 100 | 4yboC | 100 | 4uhpE | 100 | 5d9kA | 100 | 5cysA | 100 |
| **Average** | **95.05** |  | **57.22** |  | **80.56** |  | **89.11** |  | **94.57** |

**Table S3:** STARPDB webserver annotation accuracy of ligand and metal modules.

| **Ligand** | | | **Metal** | | |
| --- | --- | --- | --- | --- | --- |
| **PDBID** | **Ligand name** | **Accuracy** | **PDBID** | **Metal name** | **Accuracy** |
| 3jakK | GTP | 20.83 | 2n6aA | CA | 79.17 |
| 3jarI | GDP | 46.15 | 4yw9A | MN | 41.67 |
| 4zrmA | NAD | 96.3 | 4zmcH | FE | 71.43 |
| 4wefB | NAG | 50 | 5aw7A | MG | 100 |
| 4wybI | ATP | 21.74 | 5bv9A | CA | 0 |
| 5d8xR | HEM | 91.67 | 5c4aI | ZN | 87.5 |
| 5depE | PO4 | 0 | 5ch9A | CO | 87.5 |
| 4qhuC | SO4 | 44.44 | 5dhkA | MG | 100 |
| 4v0zA | PEG | 25 | 4uahA | CU | 100 |
| 5acoH | MAN | 14.29 | 4xfwA | ZN | 33.33 |
| **Average** |  | **41.04** |  |  | **70.06** |
